# Genomic Selection and Use of Molecular Tools in Breeding Programs for Indigenous and Crossbred Cattle in Developing Countries: Current Status and Future Prospects

**DOI:** 10.3389/fgene.2018.00694

**Published:** 2019-01-09

**Authors:** Raphael Mrode, Julie M. K Ojango, A. M. Okeyo, Joram M. Mwacharo

**Affiliations:** ^1^Animal Biosciences, International Livestock Research Institute, Nairobi, Kenya; ^2^Animal and Veterinary Science, Scotland Rural College, Edinburgh, United Kingdom; ^3^Small Ruminant Genomics, International Centre for Agricultural Research in the Dry Areas (ICARDA), Addis Ababa, Ethiopia

**Keywords:** genomic selection, indicus cattle, GBLUP, sexed semen, accuracy

## Abstract

Genomic selection (GS) has resulted in rapid rates of genetic gains especially in dairy cattle in developed countries resulting in a higher proportion of genomically proven young bulls being used in breeding. This success has been undergirded by well-established conventional genetic evaluation systems. Here, the status of GS in terms of the structure of the reference and validation populations, response variables, genomic prediction models, validation methods, and imputation efficiency in breeding programs of developing countries, where smallholder systems predominate and the basic components for conventional breeding are mostly lacking is examined. Also, the application of genomic tools and identification of genome-wide signatures of selection is reviewed. The studies on genomic prediction in developing countries are mostly in dairy and beef cattle usually with small reference populations (500–3,000 animals) and are mostly cows. The input variables tended to be pre-corrected phenotypic records and the small reference populations has made implementation of various Bayesian methods feasible in addition to GBLUP. Multi-trait single-step has been used to incorporate genomic information from foreign bulls, thus GS in developing countries would benefit from collaborations with developed countries, as many dairy sires used are from developed countries where they may have been genotyped and phenotyped. Cross validation approaches have been implemented in most studies resulting in accuracies of 0.20–0.60. Genotyping animals with a mixture of HD and LD chips, followed by imputation to the HD have been implemented with imputation accuracies of 0.74–0.99 reported. This increases the prospects of reducing genotyping costs and hence the cost-effectiveness of GS. Next-generation sequencing and associated technologies have allowed the determination of breed composition, parent verification, genome diversity, and genome-wide selection sweeps. This information can be incorporated into breeding programs aiming to utilize GS. Cost-effective GS in beef cattle in developing countries may involve usage of reproductive technologies (AI and *in-vitro* fertilization) to efficiently propagate superior genetics from the genomics pipeline. For dairy cattle, sexed semen of genomically proven young bulls could substantially improve profitability thus increase prospects of small holder farmers buying-in into genomic breeding programs.

## Introduction

Genomic selection (GS) has resulted in rapid rates of genetic gains especially in dairy cattle in developed countries with the consequence that a higher number of currently artificial insemination (AI) active sires are genomically proven young bulls in the USA (Hutchison et al., 2014). The authors reported that young bulls accounted for 28 and 25% of Holstein and Jersey inseminations in 2007, respectively. These percentages increased to 51 and 52%, respectively, in 2012 due to the use of genomically proven young bulls. Well-established conventional genetic evaluation systems have provided the strong foundation for the success of GS in these countries. Furthermore, the existence of well-developed breeding structures, particularly breeding companies, has made enormous contribution to the success. In the dairy and beef industry, for example, the genotyping infrastructure for bulls and associated costs has mainly been undertaken by AI companies such as CRV in the Netherlands (https://www.crv4all.com/), ABS in the USA (http://www.absglobal.com/us/) and Semex in Canada (http://www.semex.com/). In addition, these companies provide an efficient system for delivering superior genetics from the genomics pipeline.

In developing countries especially in Africa and Asia, most of the production occurs in small holder systems which are characterized by small herd sizes, lack of performance, and pedigree recording and therefore, the non-existence of conventional genetic evaluation systems (Kosgey and Okeyo, 2007). However, in some countries like Brazil in Latin America, the existence of breed associations have resulted in the establishment of some degree of data and pedigree recording and genetic evaluation (Silva et al., 2016; Boison et al., 2017), but there is still the lack of breeding structures such as AI companies, to drive breed improvement programs. Therefore in the era of genomics, most genotyping activities in developing countries are undertaken by breed organizations or associations, such as in Brazil (Carvalheiro, 2014; Silva et al., 2016), or are a result of several development projects, such as the East Africa Dairy Development Project (Brown et al., 2016), and the African Dairy Genetic Gains Cattle project (https://www.ilri.org/node/40458). Consequently, the number of genotyped animals tend to be limited; are mostly females, and this has major influence on both the size and structure of the reference and validation populations.

Given these characteristics, this paper examines the current status of GS and use of molecular tools in breeding programs for dairy and beef cattle in developing countries and offers some future perspectives. The basic principle of GS is that single nucleotide polymorphisms (SNPs) are assumed to be at linkage disequilibrium (LD) with QTLs in the genome. Therefore, the use of SNPs as markers enables all QTLs in the genome to be indirectly identified through the mapping of chromosome segments defined by adjacent SNPs. The implementation of GS usually involves estimating the SNP effects in a reference population which consists of individuals with phenotypic records and genotypes. This is then followed by prediction of genomic estimated breeding values (GEBV) for selection candidates (validation data set) with no phenotypes of their own (Meuwissen et al., 2001). Therefore, the current status of GS in developing countries is presented under the broad subtitles of the stages involved in the implementation of GS such as structure of the reference and validation populations, definition of input variables, genomic prediction models, validation methods, imputation efficiency, genotyping strategies, and routine genomic evaluation. A section on the use of molecular genomic tools and identification of genome-wide signatures of selection is then presented.

## Structure of the Reference and Validation Populations

As indicated earlier, the lack of major AI companies to drive the initial breed improvement and genotyping activities in developing countries has meant smaller number of animals are genotyped and most of these are females. Firstly, it becomes very difficult to clearly define separate reference and validation populations, consequently studies have been designed to optimally use the available information. In general, these reported studies on genomic prediction in dairy and beef cattle are characterized by small reference populations (500–3,000 animals, Table 1) and most validations are undertaken in test data sets created by either random or structured sampling from all genotyped animals. A few of these reference populations are a combination of both bulls and cows (Boison et al., 2017) but most are cows (Brown et al., 2016; Silva et al., 2016). This has implications in terms of the accuracy of genomic prediction, which has tended to be lower compared to those obtained in developed countries, given the limited information of the response variable when using cow records.

**Table 1:** Summary of genomic selection studies in developing countries.

**References**	**Breed**	**Traits studied**	**Chip size**	**Population size**	**Reference population**	**Size of validation set**	**Models**	**Response variable**	**Accuracy of prediction**	**Regression coefficients (response variable on predictions)**
Boddhireddy et al. (2014)	Nellore cattle	22 traits composed of reproductive, productive, visual body conformation scores traits	Illumina BovineHD BovineSNP50 (50 K) Chip	2,241	1793	448 (5-fold cross validation)	BayesC	EBVs	0.34–0.58	0.40–0.87
Boison et al. (2017)	Gyr (Bos indicus) dairy cattle	Milk, fat and protein yields and Age at first calving	Illumina BovineHD (Bulls) BovineSNP50 (50 K) chip (cows)	464 bulls 1688 cows	264–281 bulls only or plus 1177–1597 cows	115–152 Bulls	GBLUP	dEBVs	0.46–0.56 (only bulls reference) 0.47–0.62 (bull+cow reference)	0.73–0.97 (Bulls only or Bulls plus cows in reference)
Brown et al. (2016)	East Africa Crossbred cattle	Milk yield	Illumina Bovine HD	1038 cows	565–835 cows	178–448 cows	GBLUP and BayesC	Corrected phenotypes	0.32–0.41 (GBLUP) and 0.28–0.35 (BayesC)
Cardoso et al. (2014)	Bradford and Hereford cattle	Tick resistance	Illumina 50 K (cows) and Illumina HD (sires)	113 sires 3545 cows	2765	691 (5-fold cross- validation)	GBLUP, BayesB and ssGBLUP	dEBV	0.38–0.48 vaidation set by K–means clutsering 0.40–0.60 (validation set by random clustering	0.29–1.46 (K-M) 0.25–0.83 (Random)
Costa et al. (2014)	Nellore Cattle	Heifer rebreeding Age at first calving, and Early pregnancy occurrence	Illumina HD	2,056 females	1,853	185 (10-fold cross)	GBLUP, BayesCπ and IBLASSO	dEBV and Corrected Phenotypes	0.29–0.54 (GBLUP) 0.34–0.57 (BayesCπ) and 0.37–0.58 (IBLASSO)	0.84–0.88 (GBLUP) 0.89–1.14 (BayesCπ)) and 0.81–0.87 (IBLASSO)
Fernandes Júnior et al. (2016)	Nellore Cattle	REA, BFT and HCW	Illumina HD	1756 Nellore steers	1405	351 (5-fold cross-validation)	Bayesian ridge regression (BRR), BayesC (BC) and Bayesian Lasso (BL)	Corrected phenotypes and EBVs	0.21–0.46 (BRR) 0.23–0.46 (BC) 0.22–0.47 (BL)	0.40–0.99 (BRR) 0.37–0.93 (BC) 0.39–1.02 (BL)
Neves et al. (2014)	Nellore Cattle	15 Economically important traits	Illumina HD	691 Bulls	307–494	115–187	GBLUP, BayesC, BLASSO	dEBV	0.17–0.72 (GBLUP) 0.20–0.69 (BayesC) 0.19–0.74 (BLASSO)	0.75–1.24 (GBLUP) 1.01–2.35 (BayesC) 0.91–2.17 (BLASSO)
Silva et al. (2016)	Nellore beef cattle	RFI, FCR, ADG and DMI	Illumina HD	788 Cows	424–617	91–337	ssGBLUP, GBLUP and BayesCπ	Corrected phenotypes and EBVs	0.23–0.48 (GBLUP) 0.23–0.48 (BayesCπ) 0.30–0.49 (SSGBLUP)	0.78–0.90 (GBLUP) 0.05–3.10 (BayesCπ) 0.75–1.16 (ssGBLUP)
Terakado et al. (2014)	Nellore Cattle	Weight Gain Birth to weaning (GBW) and weaning to Yearling (BWY)	Illumina HD	1,658 females and 1,002 males	2118 (GBW) 988 (GWY)	531 (GBW) 246 (GWY)	GBLUP	dEBV	0.21–0.48 (GBW) 0.21–0.28 (GWY)	0.38 (GBW) 0.25 (GWY)

*EBVs, Estimated breeding values; dEBV, de-regressed breeding values; IBLASSO, Improved Bayesian LASSO*.

However, the inclusion of cows in the reference population has resulted in up to 5-fold increase in the size of the reference population in some cases and increases of up to 12% in accuracy compared to using only bulls (Boison et al., 2017). In some of the studies (Neves et al., 2014; Silva et al., 2016; Boison et al., 2017), the accuracy of genomic prediction was undertaken in validation sets consisting of young bulls born in more recent years. Thus, the accuracy of genomic prediction was evaluated in future selection candidates (forward validation) and thus better reflect the accuracy that will be obtained when selecting young animals based only on their genotypes.

In the other studies, validation sets were created from all genotyped animals by either random or structured sampling such as clustering (Ding and He, 2004) or sampling based on genomic relationship matrix (Cardoso et al., 2014) or breed composition (Brown et al., 2016). In such cross validation studies, the validation sets tend to be contemporaneous to the reference animals to some degree. Thus, the extent to which such estimates of accuracy are realized when selecting younger animals for breeding will be influenced by the degree of the relationship between the reference and the sampled validation sets. Thus, cross validation may not necessarily give the best results in terms of predicting the accuracy of selecting the youngest animals for breeding.

The influence of the relationships between various validation sets derived by sampling and the reference set on the accuracy of genomic prediction has been examined in a few studies. Boison et al. (2017) observed that the average genomic relationship for the five top individuals with highest relationships in the reference and validation data sets varied from 0.321 to 0.410. Corresponding ranges of estimates considering the top 10 individuals were 0.262–0.362. These values were higher than estimates reported in other populations (Clark et al., 2012; Neves et al., 2014). Boison et al. (2017) indicated an increase of 0.1 in the average genomic relationship for the top five individuals in the reference and validation sets (roughly equivalent to adding the sire of a selection candidate to the reference population), resulted in a substantial increase in accuracy of prediction by about 0.05. Similarly, Fernandes Júnior et al. (2016) also used the genomic relationship matrix to examine the relationship between the reference and 5-fold validation sets. The average of the maximum relationship was equal to about 0.25 and the average for the top five and 10 individuals with highest genomic relationships were 0.19 and 0.17, respectively. These values are much lower than those reported by Boison et al. (2017) and approximately correspond to the average value of 0.125 for distant relationships computed from pedigree information by Clark et al. (2012). However, Silva et al. (2016) examined the relationship between the reference animals and three sampled validation sets (random, young, unrelated) using the pedigree relationship matrix. The random had the highest relationship between the reference and validation sets, with 2.14% of the animals having relationship coefficients ranging from 0.25 to 0.50 in both datasets. Corresponding estimates were 1.17 and 0.53% for the young and unrelated validation sets, respectively. As expected, the mean accuracy of genomic predictions reported by Silva et al. (2016) from young validation set was intermediate to those for the unrelated and random data sets, with the latter being the highest.

Clark et al. (2012) indicated that the best predictor of accuracy was an animal's mean top 10 relationships with the reference followed by its highest relationship to the reference. Habier et al. (2010) reported that maximum relationship values of 0.6–0.49 between reference and validation sets gave the best estimates of accuracy of predictions. In general, the relationship between the training and validation sets in the genomic prediction models implemented in developing countries will fall within the categories of close relationships (0.5) and distant relationships (0.125) (Clark et al., 2012).

The small reference population call for collaboration between developed and developing countries, given that some of the sires used in the latter could have been imported from the former. The benefits of including foreign genotypes in estimating accuracy of genomic prediction for milk, fat, and protein yields in Brazil Holstein was examined by Li et al. (2015) by including information from Nordic and French Holsteins. None of the Brazilian bulls and cows were genotyped, but a bivariate ssGBLUP approach was implemented incorporating genotypes of 5,244 and 5,088 Nordic and French bulls, respectively, that were genotyped with Illumina 50 K chip and their de-regressed breeding values (dEBVs) expressed in a Nordic scale. The first lactation yield of the Brazilian cows expressed in 305-day yields was used in the analysis with 115 of the Nordic and 19 of the French bulls represented as sires of these cows. The inclusion of only the Nordic sires resulted in increases in sire accuracies from a cross validation approach of 13, 64, and 4% for milk, fat, and protein yields, respectively, from the genomic prediction compared to using the pedigree relationship matrix. Including both French and Nordic bulls resulted in increases of 2 and 45% in reliability for milk and fat, respectively, but none for protein. While the expression of the dEBVs for French bulls in the Nordic scale simplified the analysis to a bivariate model, it could have limited the realization of all possible benefits of including information from the French bulls. However, the increases in cow reliabilities from using foreign genotypes were rather marginal. While the study demonstrated possible benefits from incorporating foreign genotypes especially for the Brazilian bulls, it also stressed the need to undertake some genotyping in the developing countries especially if the accuracy of cow evaluations is to increase substantially. Similarly, Haile-Mariam et al. (2015) demonstrated the benefits of incorporating foreign information in the genomic prediction for the Jersey breed which has a small reference population of about 784 Australian bulls. The inclusion of about 2,000 foreign bulls with only daughter information in the Netherlands and New Zealand increased the genomic accuracy by 5% on average across 6 main dairy traits in the validation bulls relative to the use of only Australia information. The increase in accuracy resulting from the use of bulls with foreign information was relatively higher when bulls and cows in the validation sets were less related to the reference set.

The small reference populations indicate the need for across regional genomic prediction systems where this is possible with data pooled across nearby countries especially in sub-Sahara Africa, where dairy systems tend to be similar. Several procedures and approaches for combining data across breeding programs or countries have been developed and these range from post-evaluation blending procedures, application of appropriate linear models, or Bayesian methods (Vandenplas and Gengler, 2015; Vandenplas et al., 2018). Mrode et al. (2018) analyzed pooled data for milk yield from crossbred cattle in Kenya and Tanzania. The number of cows with genotypes in Tanzania was 539 and in Kenya there were 1,034. The joint genomic prediction resulted in increased accuracy of genomic prediction in Tanzania by more than 20% for most categories of cows with substantial improvement of the predictive ability of the model for Tanzania. However, there was no much gain in accuracy for Kenyan animals from the joint analysis compared to the within country analysis as the Tanzania data was very limited and the average relationship between both populations was rather low.

## Genomic Prediction Models and Response Variables

The large data sets of genotyped bulls available for dairy cattle in the developed countries has influenced the choice of models implemented for genomic prediction in developing countries. In addition, the complex models such as the random regression models in dairy cattle and multi-trait models in beef cattle implemented for the conventional genetic evaluation at the national level in most developed countries has given birth to the two-step genomic prediction systems especially for dairy cattle (http://www.interbull.org/ib/nationalgenoforms). This implies the running of conventional evaluations to compute EBVs, which are subsequently de-regressed (dEBV) and used as input variables for SNP-BLUP or GBLUP genomic predictions (http://www.interbull.org/ib/nationalgenoforms). Recently, some developed countries have implemented single-step genomic evaluations, ssGBLUP, mostly in beef cattle (Moore et al., 2018) for the evaluation of fertility and calf traits.

However, in developing countries, the small data set of genotyped individuals, in addition to either no or less complicated conventional genetic evaluation systems, have resulted in the implementation of GBLUP and various Bayesian methods and a summary is presented in Table 1. GBLUP has been commonly utilized with **G** usually computed by method 1 of VanRaden (2008). Importantly, the computation of **G** has enabled the estimation of genetic relationship between different groups of animals and to undertake genetic evaluations in the absence of pedigree information (Mrode et al., 2018). The availability of genotypic information on only a limited proportion of animals has promoted the implementation of ssGBLUP (Misztal et al., 2009) enabling the combination of pedigree and genotypic information in the prediction of the genetic merit, usually resulting in higher accuracy due to the utilization of all available data (Cardoso et al., 2014; Silva et al., 2016). Plurality of Bayesian methods (Table 1) have been utilized, possibly due to the limited data size. However, no clear advantage of these methods over GBLUP or ssGBLUP have been demonstrated. It could be inferred that developing countries do not lag behind in terms of models used in predicting genomic genetic merit compared to developed countries.

The availability of genotypic data, mostly of females, have influenced the response variable used in genomic prediction models in developing countries. Most studies have therefore used corrected phenotypic records of genotyped cows as input variables for genomic prediction (Brown et al., 2016; Fernandes Júnior et al., 2016; Silva et al., 2016). This usually involves an initial genetic evaluation either using the pedigree or the genomic relationship matrix to obtain the fixed effects solutions for adjusting the phenotypic records. In some cases, phenotypic information available on each cow is variable and in some cases, weights are computed to account for the varying accuracy associated with each record (Brown et al., 2016). Like developed countries, dEBVs from conventional genetic evaluations have been used as response variables in genomic prediction (Cardoso et al., 2014; Boison et al., 2017). The dEBVs in the study of Boison et al. (2017) were weighted in the analysis based on the reliability of the dEBV and heritability of the trait. In some studies, due to limited information resulting in poor de-regression (Morota et al., 2014), EBVs have been used as response variables (Table 1); and in most of these studies the use of EBV have resulted in lower accuracy of genomic prediction compared to the use of adjusted phenotypes (Fernandes Júnior et al., 2016; Silva et al., 2016). The use of EBVs as the response is rarely the case in developed countries but the tendency is to use dEBVs especially for traits that have well-established conventional evaluations. In some cases, especially for novel traits or difficult to measure traits which are recorded mostly on cows such as feed intake, cow phenotypes have been utilized (de Haas et al., 2014).

Ideally the dEBVs used as response for the genomic prediction in the reference should not include information from the validation data set, otherwise the contribution of the information from the validation animals could lead to inflated estimates of reliabilities. However, this could not be achieved in the study by Boison et al. (2017) and so estimates of reliabilities were reported to be inflated.

## Accuracy of Genomic Predictions

Generally, the accuracy of genomic prediction is usually based mostly on correlations between the direct genomic breeding and the dEBV or adjusted phenotypes in the validation data set. When adjusted phenotype is used, the correlation coefficient is divided by the square root of the heritability of the trait to measure the correlation between predicted and true breeding values (Legarra et al., 2008; Pryce et al., 2012). Similar approach has been employed in most of the studies in developing countries (Brown et al., 2016; Silva et al., 2016). However, in the studies of Terakado et al. (2014) and Boison et al. (2017), the estimation of accuracy of genomic prediction was based on prediction error variances estimated from the inverse of the mixed model equations. This is usually termed as the theoretical or expected estimates of accuracy (VanRaden, 2008) and usually tend to be higher than the estimates obtained from correlations because it ignores changes in genetic variance due to drift or selection (Gorjanc et al., 2015). Further, the theoretical accuracy is based on the assumption that the used statistical model is the true genetic model. Taken together, the theoretical accuracies may often be inflated. The large number of animals in the reference population in the genomic prediction systems of many developed countries implies it is not feasible to obtain the inverse of the mixed model equations, hence theoretical estimates of accuracy are not usually computed routinely but it has been implemented in Canada based on a reduced set of SNPs (http://www.interbull.org/ib/nationalgenoforms).

The accuracy of genetic prediction in dairy traits ranges from 0.50 to 0.85 for production traits with medium to high heritability to about 0.20–0.50 for fertility and survival traits with lower heritability in developed countries (Moser et al., 2010; Wiggans et al., 2017). Those for beef traits are slightly lower (0.33–0.55) due mainly to lower reference population sizes (Saatchi et al., 2011; Lu et al., 2016). In the case of developing countries, the accuracies of genomic predictions have rather been low to medium in the range of 0.21–0.60. The major factors for these ranges include the small size of the reference populations and the composition in terms of being mostly cows that have lower accuracy of phenotype data than progeny tested bulls in developed countries. The deterministic prediction equations for genomic accuracy by Goddard (2009) and Daetwyler et al. (2013) could help explain such lower accuracies arising from having mainly cows in the reference population. Assuming traits are influenced by a large number of QTL, Daetwyler et al. (2013) gave the following formula to predict genomic prediction accuracy defined as the Pearson correlation (r) of true and predicted observed values: r = √ [Nph^2^ (Nph^2^ + Me)^−1^], where Np is the number of individuals with phenotypes and genotypes in the reference population, h^2^ is the heritability of the trait or reliability of breeding values in the reference population, and Me is the number of independent chromosome segments. Me can be computed as Me = 2NeL, with Ne equals the effective population size and L, the genome length in morgans. Using typical values of 100 and 30 for Ne and L, respectively, (Daetwyler, 2009), and assuming Np of 1,000, reliabilities of about 0.80 and 0.3 for de-regressed breeding values (dEBV) for progeny tested bulls and individual cows, respectively, the formula indicates that about 4–5 cows would be needed to provide equivalent information to one progeny tested bull. Compared to specialized dairy breeds in developed countries, effective population is likely to higher in indigenous dairy cattle and crossbreds reared in smallholder systems. Increasing the value of Ne to 200 in the above formula to account for this, indicates that the ratio of about 4 to 5 cows providing equivalent information to one bull still holds.

Also, the lack of a proper breeding program in most developing countries does not provide the breeding structure to ensure that good relationship between younger animals in the validation set are well-related to animals in the reference population. The levels of accuracy reported in most of the studies are however higher than would be obtained from the parental average although they are lower than those estimated in developed countries, thus providing a basis for the selection of good bulls that can be used as parents for the next generation.

Similar to the accuracy of genomic predictions, the regression of the response variable on direct genomic breeding values in the validation set as a measure of the calibration (inflation or deflation) of GEBV, have showed great variation (Table 1). In some of the studies, the regression coefficients were in general close to 1 as expected for traits of higher heritability except for lowly heritable traits, which, in most analysis, were over 1, meaning that predictions were underestimated (Fernandes Júnior et al., 2016; Silva et al., 2016; Boison et al., 2017). The Bayesian methods (BayesC, BayesCπ, and Bayesian Lasso) have resulted in underestimated predictions compared to GBLUP in several of these studies (Neves et al., 2014; Boison et al., 2017). However, some of these regression coefficients were rather low and below 0.5 (Table 1) and due mainly to the smaller size of the reference population. An improvement in the calibration is expected as more animals are genotyped.

## Utilizing Low Density Chip and Imputation

A major issue with the implementation of GS is the cost of genotyping and it constitutes one of the obstacles to GS in developing countries. Several studies have therefore examined the use of cheaper low-density Chips or investigated the use of low numbers of SNPs accompanied by imputation on the accuracy of genomic prediction.

Boison et al. (2017) examined the use of several LD chips, using common SNPs between the HD and the Illumina 50K, GeneSeek super genomic profiler (SGGP-20Ki), and GeneSeek genomic profiler (GGP-75Ki) in genomic prediction. The accuracy of genomic prediction they reported when only bulls were used in the reference population was similar in the LD chips compared to the HD. However, with a larger reference population consisting of bulls and cows, they reported an average increase in reliability of 3.3% across all traits with the HD marker panel compared with SGGP-20Ki. In addition, Boison et al. (2017) examined the impact of using un-imputed HD genotypes in the validation datasets compared to the use of HD genotypes imputed from LD chips. The imputation accuracy was high (about 0.96 on average) and the use of imputed genotypes had no effect on the accuracy of estimates.

Aliloo et al. (2018) investigated the efficacy of imputation in East Africa crossbred dairy cattle in terms of its impact on the accuracy of imputation and genomic prediction using four different commercial chips [Illumina BovineLD v2, BovineSNP50 v3, GeneSeek-Genomic-Profiler (GGP) Bovine 50 K, and Indicus 35 k v1.03 (Neogen Corporation, Lincoln, NE, USA)] with different reference populations and three different imputation algorithms [FIimpute v2.2; (Sargolzaei et al., 2014), Beagle v4.1 (Browning and Browning, 2016), and Minimac v3 (Das et al., 2016)]. The highest imputation accuracy was obtained with a reference population consisting of a mixture of crossbred and ancestral purebred animals and using Minimac. The accuracies of imputation, measured as the correlation between real and imputed genotypes, were around 0.76 and 0.94 for 7 and 40 K SNPs, respectively, when imputed up to a 770 K panel. In general, the accuracies of the imputation from LD chips to HD genotypes were higher as the genomic relationships increase between target and reference animals.

In addition to examining the efficiency of imputation from different commercial chips, the study of Aliloo et al. (2018) also examined the efficiency of several methods for creating low density SNP chip panels of varying sizes (3,757 to 37,8216) from the HD Illumina chip. The methods examined for SNP selection included using MAF within intervals, random selection within intervals, random selection across chromosome, MAF across chromosome and the covariance method (it accounted for the covariance between adjacent SNPs and the MAF of SNPs). The efficiency of each method was determined by the accuracy of imputing the created LD chips to the HD and the accuracy of using the imputed HD in genomic prediction. The covariance method performed best compared to various other methods. The accuracies of imputation from 7 to 40 K panels selected using the covariance method were around 0.80 and 0.94, respectively. It also resulted in higher accuracy of genomic prediction at lower densities of selected SNPs.

The influence of foreign genotypes on imputation accuracy when imputing from 6, 9, 50, and 77 K chips to 45 K markers used in the USA genomic evaluations in 2014, was examined by García-Ruiz et al. (2014) in Mexican Holstein under three scenarios: (i) using only 2,018 Mexican genotyped animals; (ii) animals from scenario (i) plus 886 related North American animals; and (iii) animals from scenario (i) and 338,073 North American genotyped animals. High imputation accuracies were obtained (96, 96, 99, and 99%, when imputing from 6, 9, 50, and 77 K chips, respectively) when using only local genotypes [scenario (i)]. With scenario (ii), the imputation accuracy increased by almost 1% for 6 and 9 K chips and half a percentage point for the 77 K chip. Comparing results with scenario (i), there was an increase of ~2% for 6 and 9 K chips, and 1% point for the 77 K chip under scenario (iii). However, no increase in accuracy was observed for the 50 K chip in any scenario because of the small number of SNPs that actually were imputed due to the large number of SNPs common in both chips. Generally, high imputation accuracies have been reported in developing countries although the reference populations are smaller compared to the ones in developed countries. This may be due to the fact that the imputation involves mostly cows and the limited number of sires may be used in these populations and hence higher degree of relatedness. However, collaboration between developed and developing countries could be beneficial in terms of further increasing imputation accuracies (García-Ruiz et al., 2014).

A purpose-built LD SNP chip for the purpose of GS in cross bred populations (Hidalgo et al., 2016) has recently been developed by the National Dairy Development Board (NDDB) of India (https://www.nddb.coop/services/animalbreeding/geneticimprovement/genomic). The SNP chip called the INDUSCHIP, consisting of 45,700 SNPs, has been developed from HD genotypes of mostly four indicus breeds (Gir, Sahiwal, Kankrej, Red Sindhi) and their taurine crosses mostly with Holstein and Jersey, in India and has been employed for the determination of breed composition and genomic prediction for milk yield.

## Routine Genomic Evaluations

The basis of tremendous genetic progress from GS in developed countries has been underpinned by routine genomic predictions several times in a year. Although several studies have been undertaken in several breeds (see Table 1) in developing countries, routine genomic prediction is undertaken in only a few breeds. Several parallel breeding improvement programs exist in the Nellore beef cattle in Brazil and some GS is currently being undertaken in some of these breeding programs (Carvalheiro, 2014). The author indicated that several independent Nellore breeding programs have already developed prediction equations for usual and difficult/expensive to measure traits, however some of the programs are using genomic predictions more as a marketing than a selection tool. Carvalheiro (2014) summarized the two business models driving GS in the Nellore cattle. In the first scenario, the breeders or the breeding programs do not have access to the genotypes and genomic prediction equations are regarded as intellectual property of the multinational private companies that invested in their development. Under this model the genomic breeding values (GEBVs) are produced, for example, by combining genomic predictions and regular EBVs as correlated traits in a multi-trait mixed animal model analyses (Garrick, 2011). Therefore, the breeding programs become dependent on the company that sells the GEBVs and its sustainability depends on the interest of the commercial company to constantly invest in recalibrating the prediction equations. The second model he described involves breeding programs and the breeders have full access to the genotypes. He considered this a very attractive model because no dependencies exist between any two segments, enabling breeding programs to change their service providers without any prejudice if they are not satisfied, for example, with the genotyping cost or with the quality of the genetic evaluations.

The Africa Dairy Genetic Gains (ADGG) project in Tanzania and Ethiopia are currently establishing a pipeline for routine genetic evaluation using the genomic relationship matrix, in addition to screening and selecting young bulls using the genomic predictions (https://www.slideshare.net/ILRI/mrode-wcap). The non-existence of AI companies to drive genetic improvement programs, implies that genetic and genomic evaluations would inevitably be linked to either National Artificial Insemination Centers or breed societies to help deliver the superior genetics. This is the current approach being exploited by ADGG while encouraging public-private partnership in the space. The beef breeds in South Africa are in the process of implementing GS but current activities are still limited to defining the reference population and understanding the population structure.

## Molecular Genomic Tools and Identification of Genome-Wide Signatures of Selection

Genome sequencing and SNP genotyping technologies, and new statistical tools have prompted a transition from studies focusing on the analysis of neutral variation to functional variation. These developments have led to new tools for addressing fundamental and applied questions in evolutionary and developmental biology, and animal breeding. Sequencing of full genomes and the development of SNP Chip sets has led to studies on identification and mapping of genes and QTLs, genome-wide association analysis (GWAS) and genome-wide signatures of selection, introgression, and/or admixture. The studies have led to the identification of many genes and some incorporated into selection schemes. In developed countries, whole genome sequence analysis and GS are being applied in breeding schemes of major food animals (cattle, sheep, goats, chicken, pigs). In developing countries, genomic technologies are applied to assessing genetic diversity and admixture and signatures of selection to identify genomic regions and variants contributing to variation.

Genomic technologies have shown that indigenous cattle in developing countries have high levels of genome diversity compared to commercial breeds (Kim et al., 2017) due to their different breeding history (Freeman et al., 2004; Decker et al., 2014; Flori et al., 2014; Edea et al., 2015). Kim et al. (2017) also revealed the genomes of indigenous breeds are admixed which suggests genomic diversity as an efficient adaptation strategy. SNP genotyping and whole-genome sequencing has shown the genome admixture is of ancient and recent origin. An analysis of zebu cattle from Kenya, Uganda, and Nigeria revealed an even admixed autosomal Asiatic indicine^*^African taurine genome composition as well as European taurine ancestry (Mbole-Kariuki et al., 2014; Bahbahani et al., 2017) confirming previous findings (Hanotte et al., 2002; Decker et al., 2014). The Asian indicine^*^African taurine composition is ancient and decreases westwards and southwards from the Horn of Africa (Hanotte et al., 2002; Decker et al., 2014) while the European taurine background arises from recent crossbreeding of local cattle with European *Bos taurus* breeds. For example, the Borgou cattle of West Africa is a stabilized admixed breed with genetic contributions from four African taurine (Baoulé, Somba, Lagune, N'Dama) and two African Zebu (Fulani, Bororo) cattle, whose origin traces back to about 130 years ago (Flori et al., 2014). The genomes of Kenyan local cattle have contributions from several *B. taurus* breeds including Guernsey, Norwegian Red, and Holstein with the contribution of Holstein-Friesians being the most substantial (Kim and Rothschild, 2014). The authors postulate the admixture to have occurred in recent times. Admixed genomes are also a common feature of indigenous and locally developed breeds of cattle in South Africa (Makina et al., 2014). Admixed genomes have also been observed in Asian (India, Pakistan, China, and Indonesia) *Bos indicus* cattle which show evidence of *Bos javanicus* ancestry (Decker et al., 2014). Kumar et al. (2003) reported an ancestral influence from taurine cattle in South Asian *Bos indicus* cattle, probably of Near eastern origin and Wangkumhang et al. (2015) observed a Southeast Asian indicine ancestry in the genomes of Thailand cattle.

Written pedigree records are lacking in most small holder farms in developing countries which makes it almost impossible to make informed breeding decisions. Genomic technologies can be valuable in this case in assessing breed composition and parentage assignment (Werner et al., 2004; Weerasinghe, 2014). Recently, Strucken et al. (2017) demonstrated such an application using crossbred cattle in East Africa (Kenya, Uganda, Ethiopia, Tanzania). The authors identified two marker panels with 200 SNPs each. One panel predicted best, the dairy breed compositions and the other resulted in accurate estimates of parentage assignment. A composite panel incorporating the 400 SNPs achieved sufficient accuracy in estimating breed admixture proportions but not parentage identification.

The development of new technologies which assess genome architecture with high resolution (full genome sequences, HD Chips etc.) has resulted in a large number of studies investigating genome-wide signatures of selection in indigenous cattle in developing countries and especially in African cattle. For instance, 18 candidate regions under selection and intersecting genes and QTLs associated with production and reproduction performance and adaptation to environmental stress (e.g., immunity and heat stress) were identified in East Africa cattle from the analysis of SNP genotype datasets (Bahbahani et al., 2017). Bahbahani et al. (2018) found several dairy trait QTLs overlapping candidate selection regions in Kenana and Butana cattle based on the analysis of SNP genotype data. Using whole genome scans, Gautier et al. (2009) identified 53 genomic regions that spanned 42 genes with functions related to immune response, nervous system and skin, and hair properties in West African cattle. Makina et al. (2015) identified 47 candidate selection regions which also spanned genes associated with adaptation to tropical environments, nervous system, immune response, production and reproductive performance in South African cattle. In a study that analyzed genome sequences of indigenous breeds of cattle from East, West and Southern Africa, Kim et al. (2017) identified signatures of selection including genes and/or pathways controlling anemia, feeding/drinking behavior and circadian rhythm in the N'Dama, coat color and horn development in Ankole, and heat tolerance/thermoregulation and tick resistance in Boran, Ogaden, and Kenana cattle. The findings from the selection signature studies spanning genes with functions related to production, reproduction and adaptation, suggest that genomes of cattle African indigenous cattle have been uniquely selected to maximize hybrid fitness for adaptation to reproduce and perform in stressful environments.

## Future Prospects

The major factor limiting the application of GS in developing countries is poor breeding infrastructure that is fundamental to conventional breeding, lack of routine recording of reliable phenotypes and good analytical tools to synthesize the data, providing timely feedback to help improve farmer management and husbandry techniques. The ADGG has sort to address some of these major bottlenecks in East Africa by employing recent developments in information and communication technology (ICT). In addition, as Ribaut et al. (2010) indicated the revolution in ICT has also created opportunities to counter some of the shortcomings in resources through the establishment of global virtual platforms. For example, the Bill & Melinda Gates Foundation and CGIAR Generation Challenge Program has established a public molecular breeding platform (https://www.gatesfoundation.org/Media-Center/Press-Releases/2010/02/GCP-launches-Molecular-Breeding-Platform) as a one stop shop which centralizes functional access to modern breeding technologies and marker service laboratory, data management and analysis for crops. A similar initiative for livestock, or incorporating livestock requirement to such a center, will boost genomic activities and increase cost efficiency. The rapid developments in marker technologies has led to high-throughput platforms for SNP genotyping and hence reduced costs. However, in the absence of such centers as described above, good outsourced cost-effective genotyping services which are easily accessible are now available. This provides opportunities to increase the efficiency of implementing advanced genomics in developing countries.

The provision of bundled services beyond just GS will accelerate the adoption and use of molecular tools including GS. Programs for genetic improvement utilizing genomics approaches should include the development of tools for parentage verification, breed composition determination, mating tools that exploit genomic information, traceability, breed characterization, and tools for computing genomic inbreeding readily and addressing issues relating to sustainable utilization. Such approach maximizes the benefits of genotyping and increases cost-efficiency.

Generally, in the beef industry, GS is expected to generate a more modest increase in genetic gain for regular traits compared to dairy cattle partly due to the breeding structure and relatively limited use of AI. Strategies for optimizing cost-benefits for the application of GS in beef cattle in developing countries are still being investigated. Carvalheiro (2014) compared several scenarios for the application of GS in Nellore cattle using the current breeding scheme for Nellore as the base standard. This, in brief, consisted of a breeding program with half of its calves being born from AI proven bulls and the other half from natural mating sires and estimated an annual genetic gain of 0.134 genetic standard deviation for growth traits. However, when only genotyped young sires were used for a fixed time in AI, annual genetic progress increased by about 58% compared to the base situation. When a scheme that incorporated GS in addition to exploring the use of *in vitro* fertilization (IVF) (with embryos produced by genotyped donors accounting for 5% of the calves) was investigated, the annual increase in genetic gain was 79% relative to the base situation. Carvalheiro (2014) concluded, more pronounced genetic gains can be realized, if GS is applied in combination with reproductive technologies, which agrees with the observations of García-Ruiz et al. (2014). Carvalheiro (2014) further indicated that the production of embryos through IVF is becoming very accessible in Brazil, and he indicated a cost of about US$150 per calf born. This would indicate that much higher returns from the application of GS in beef cattle in developing countries would involve pronounced usage of reproductive technologies incorporating to some degree, both the widespread use of AI and IVF. Even in dairy cattle, future investments in the production of high quality genomically proven embryo for use in medium to large scale farms could be a routine for the rapid dissemination of superior genetics leading to more benefits from GS.

Another development in reproductive technologies that is more likely to have a profound effect in the dairy cattle industry is the use of sexed semen. In the small holder system, the cost of purchasing a replacement heifer constitute a major capital investment not easily affordable to most of the farmers. Also, the milking of the dairy cow constitutes the main source of income in the dairy farmer in India, given the sacred status of cattle. The use of sexed semen of genomically proven young bulls with a very high probability of a female calf, could substantially improve productivity and profitability of small holder farmers and therefore offers prospect for farmers buying-in into genomic breeding programs. Thus, continuous improvement in semen sorting technologies and methods to enhance conception rates with use of sexed semen opens up future prospects for the application of GS.

Collaboration between developing and developed countries will be important in implementing genomic breeding technologies in the former, especial in dairy cattle, where there has been a large importation of bulls. It is likely that most of these bulls have been genotyped in the developed countries and willingness to share genotypes and some other relevant performance data will help in enlarging the reference population and hence the accuracy of genomic predictions in developing countries. Some of the possible impacts have been demonstrated by Li et al. (2015).

The ability of Governments to put in place enabling policies, statutory and regulatory frameworks that encourage private-public partnerships will be crucial in the long term in sustaining breeding programs based on conventional or genomic approaches. Also, the limited genomic data in each country calls for pooling of data across multiple countries or geographic regions to maximize the benefits of GS. Initial possible increases in accuracies, the result of pooling data across two countries have been demonstrated (Mrode et al., 2018). However, pooling data across countries could be a sensitive issue in terms of who has access to the data from other countries. Thus, there is the need by different government bodies in developing countries to come up with proper and well-defined protocols that guide and govern data sharing with adequate confidentiality.

## Author Contributions

RM and JM undertook most of the work in this manuscript and contributed equally. JO and AO contributed as part of the team that generated data for the initial work on genomic prediction in East Africa and part of the current ADGG project that is stimulating GS in East Africa.

### Conflict of Interest Statement

The authors declare that the research was conducted in the absence of any commercial or financial relationships that could be construed as a potential conflict of interest.
